# Charting Regulatory Stewardship in Health Research: Making the Invisible Visible

**DOI:** 10.1017/S0963180117000664

**Published:** 2018-04

**Authors:** GRAEME T. LAURIE, EDWARD S. DOVE, AGOMONI GANGULI-MITRA, ISABEL FLETCHER, CATRIONA MCMILLAN, NAYHA SETHI, ANNIE SORBIE

**Keywords:** regulation, governance, research ethics, stewardship, custodianship, data, collective responsibility, proportionality

## Abstract

This section focuses on the ethical, legal, social, and policy questions arising from research involving human and animal subjects.

## Abstract (normal)

This article analyses a hitherto largely obscured feature of regulatory environments in health research; namely, the role of regulatory stewardship. Through examples drawn from research ethics committees, emerging technologies, and governance of research resources, it outlines the essential features of regulatory stewardship, and argues that this concept can demonstrate considerable added value for all parties in delivering and benefiting from efficient and effective navigation of regulatory landscapes. It offers an exposition of the normative principles and associated responsibilities of the concept. The extant invisibility of regulatory stewardship requires fuller recognition and better integration of the approach into the effective functioning of law and regulation in the health research context.

## Introduction

In June 2016, the High Level Expert Group (HLEG) of the European Open Science Cloud (EOSC) suggested that up to 500,000 “data experts” trained in the principles of open science and research data commons will be needed to support the more than 70,000,000 researchers and other workers dealing with data and innovation around the globe.^1^ This reflected the call of the Council of the European Union in May 2016 to the European Union Commission “to promote data stewardship—including training activities and awareness raising—and to implement Data Management Plans as an integral part of the research process.”^2^ The EOSC HLEG’s June 2016 report offered a road map to deliver on this vision, but it did not provide detail on what such a data stewardship role entails, nor on how it links with regulation in areas such as data protection and information technology.^3^ If the EOSC group’s recommendation of 500,000 data experts serving a stewardship role is an accurate measure of an unmet need, then the urgency to craft the principles and standards behind stewardship, both within and outside the data context, is considerable. More recently still, a joint report by the British Academy and the Royal Society on data management and use has called for the creation of a new stewardship body to support delivery of the full breadth of critical functions necessary for effective data governance. It argues that such a stewardship body should be: “(i) independent, (ii) deeply connected to diverse communities, (iii) expert across and beyond disciplines, (iv) tightly coupled to decision processes, (v) durable and visible, and (vi) nationally focused but globally relevant.”^4^ We suggest that, taken together, these initiatives represent a call for a new model of *regulatory stewardship*. However, if the complementarity of regulatory stewardship to existing regulatory mechanisms is not explored, defined, and defended further, we risk adding to regulatory burden rather than alleviating it.

This article builds on the recommendations from the British Academy/Royal Society and on the EOSC HLEG’s acknowledgement of the potential importance of stewardship in the research data context; it does so by charting what we believe are the key features of regulatory stewardship, including its core principles, in the context of health research. Although stewardship is a concept that has received a decent amount of treatment in policy and healthcare ethics literature in recent years, regulatory stewardship is an invisible but centrally vital concept deserving of greater recognition and instantiation in regulatory practice. We argue herein that regulatory stewardship can demonstrate considerable added value for all parties in delivering and benefiting from efficient and effective navigation of regulatory landscapes, as long as clarity is provided about the nature of its features and functions. Moreover, the potential for such a role extends across the entirety of the health research context, from data-related research to clinical trials involving investigational medicinal products.

Therefore, although we welcome initiatives such as the EOSC to bolster research and innovation, we argue that their success, especially in the health research context, rests on first adequately addressing three features with respect to regulatory stewardship:

1) the need to identify and to communicate clearly the meaning of, principles behind, and responsibilities that arise from “stewardship”;
2) the importance of recognizing, defending, and implementing “regulatory stewardship” as the collective responsibility of all stakeholders involved across all areas of health-related research (i.e., stewardship is not under the remit of regulators or researchers alone); and
3) the central role of clearly embedding appropriate regulatory stewardship responsibilities in law and regulation, including into any training and awareness raising for stakeholders in health research.

The analysis proceeds as follows: first, we provide an account of the range of ways in which stewardship is defined; second, we consider the range of ways in which stewardship is invoked in a variety of contexts; third, we offer an analysis of the nature of the roles that seem to be undertaken, and the values and principles that they seem to embody; and fourth, we argue for a model of regulatory stewardship that incorporates obligations to support and promote responsible health research in ways that are complementary to other regulatory functions. Our model is composed of key features that we outline at the end of the article.

We acknowledge that the recognition of a role for stewardship in health research assumes a position that is determinedly pro-research. We do not seek to question this assumption in this article; rather, we aim to chart the features of this increasingly common function across various contexts, and thereby contribute to completing the full picture of health research regulation. Our claim to originality is in the development of the concept of *regulatory* stewardship.

## What is Stewardship?

There is disagreement in the literature on what constitutes a steward or stewardship, in part because they can operate at different levels (from the local to the global), although common features are present. In this section, we highlight several understandings of stewardships in the healthcare policy and ethics context, with a view to pulling out common threads to develop the contours of regulatory stewardship.

The HLEG of the EOSC defines data stewardship as “the entire process that deals responsibly with one’s own and other people’s data throughout and after the scientific discovery process,”^5^ suggesting that stewardship entails diligent management of something or someone across multiple stages of an endeavor, although this does not ipso facto comprise an ethical or legal duty. The HLEG does not spell out which actors may be charged with a stewardship role, nor, as we have said, how this role aligns with regulations that impact on data processing and sharing.

The idea of stewardship is also relatively common in the field of public health. The Nuffield Council on Bioethics has used it to capture notions of state responsibility for providing conditions that allow people to be healthy, especially in relation to reducing health inequalities.^6^ Examples include measures “[to] promote health not only by providing information and advice, but also by programmes to help people overcome addictions and other unhealthy behaviours.”^7^ The framing of the role as “stewardship” is important for a number of features, not the least of which is the sense that part of the responsibility concerns an ushering and guiding role in the promotion of health, well-being and equality, while not being overly paternalistic and respecting the integrity of the surrounding infrastructure.

Somewhat similarly, the World Health Organization (WHO) defines stewardship in political rather than moral or legal terms. For them, stewardship constitutes:

the wide range of functions carried out by governments as they seek to achieve national health policy objectives.…Stewardship is a political process that involves balancing competing influences and demands. It will include: maintaining the strategic direction of policy development and implementation; detecting and correcting undesirable trends and distortions; articulating the case for health in national development; regulating the behaviour of a wide range of actors—from health care financiers to health care providers; and establishing effective accountability mechanisms. Beyond the formal health system stewardship means ensuring that other areas of government policy and legislation promote—or at least do not undermine—peoples’ health. In countries that receive significant amounts of development assistance, stewardship will be concerned with managing these resources in ways that promote national leadership, contribute to the achievement of agreed policy goals, and strengthen national management systems. While the scope for exercising stewardship functions is greatest at the national level, the concept can also cover the steering role of regional and local authorities.^8^

The WHO’s definition, which is perhaps the paradigmatic global understanding of stewardship, suggests that stewardship is about political (state) actors providing services to a population, setting and enforcing the rules of the game, and providing strategic direction for all the different actors involved. It is systems-level oversight with a view toward long-term sustainability of resources.

Closer to the clinical (and local) context, moral overtones come into play more overtly. For example, Lynn Jansen would seem to disagree with the WHO’s notion of stewardship, arguing that the contents in the definition are better seen as matters of distributive justice rather than as matters of stewardship in healthcare.^9^ As she writes: “In general, stewards are persons who are charged with the task of taking good care of that with which they have been entrusted.”^10^ Jansen defines stewardship in medicine as: “a duty that applies *in a space between* the obligations of health care providers to provide beneficent care to their patients on the one hand and the obligations of citizens to bring about and support a just health care system on the other. Seen with clear eyes, stewardship in medicine is neither a consequence of beneficent medical care nor a substitute for justice.”^11^

We will later return to Jansen’s comment that stewardship is a duty that falls into a space between the ethical duties of beneficence and justice. For now, we observe that for Jansen, stewardship has an important and distinctive place in clinical medical ethics. Namely, it is a duty for healthcare professionals to use healthcare resources and provision of healthcare *responsibly* when they and patients alike are confronted with what she calls “zones of discretion”; that is, a gray area in medical decisionmaking resulting from lack of evidence (e.g., when more than one treatment option is available and, according to the best evidence available, no one option is superior to the others).

Considerations of good stewardship direct health care providers to select the most cost-effective response among the eligible options in a zone of discretion. When they do so, they act responsibly because they act in a way that responds to the fact that health care resources are finite and that excessive costs for some leave less for others. They also act beneficently toward their patients, because they do not act in a way that is contrary to the best medical interests of their patients.^12^

In these zones of discretion, healthcare professionals are “free to rely on their hunches and personal styles to select an option.”^13^ Stewardship could also apply to scenarios in which healthcare professionals must decide whether to ration resources at the bedside, although Jansen acknowledges that this is a controversial position.

Writing in this journal in the context of elderly persons, David Thomasma endorses Joseph Fletcher’s somewhat legalistic notion that a steward is “somebody who acts on behalf of a principal. He or she is an agent who carries out the principal’s wishes.”^14^ Thomasma goes on to argue that stewardship entails one person helping another person achieve certain aims, and requires educating these stewards (or stewards-in-training) about the value dimensions of those that they may steward, and how some of these values can supervene otherwise standard practices.

Beyond the healthcare context, in 2010, the Presidential Commission for the Study of Bioethical Issues released a report on synthetic biology that addressed regulation of emerging technologies. In their report, the Commission dedicated a section to exploring a core ethical principle of “responsible stewardship.” As they define it:

The principle of responsible stewardship reflects a shared obligation among members of the domestic and global communities to act in ways that demonstrate concern for those who are not in a position to represent themselves (e.g., children and future generations) and for the environment in which future generations will flourish or suffer. Responsible stewardship recognizes the importance of citizens and their representatives thinking and acting collectively for the betterment of all.^15^

The report is an acknowledgement that responsible stewardship is an ethical principle of growing significance and recognition; like the HLEG on the EOSC, the Presidential Commission invokes the notion of responsibility in stewardship. Similar to Thomasma and Fletcher, the Presidential Commission suggests that stewardship involves a kind of responsible and future-attuned care for others and each other so as to improve our collective well-being. However, in the regulatory context, the Presidential Commission goes on to suggest that responsible stewardship entails “an ongoing process of prudent vigilance that carefully monitors, identifies, and mitigates potential and realized harms over time” and “requires clarity, coordination, and accountability across the government.”^16^

The Presidential Commission’s discussion of responsible stewardship also reflects a number of the points that we will argue subsequently; namely, the links with collective responsibility, the need for cross-sectoral action, and the avoidance of risk fetishisation (as opposed to legitimate risk mitigation). Where we depart, however, is in two areas. First is the association of stewardship as a component of citizen participation. A key component of our added value is placing stewardship firmly within the regulatory context. Wider deliberative democratic input has its merits, but in the regulatory context, it is specifically (and, we claim, only) the actors situated in the regulatory spaces who need to embrace the responsibility of regulatory stewardship. Second, although it is acceptable to frame stewardship in an operational way (i.e., “What can and should we, as a society, do in response to emerging technologies or a particular ‘bad’ to be responsible stewards of nature, human health and well-being, and the world’s safety, now and into the future?”), this has the potential to be set up as demanding top-down intergovernmental agency solutions. On the contrary, we ask what stewardship might mean in the health research regulatory context specifically. Further, the solutions we propose when framing stewardship in an operational way suggest a role for top-down government; however, it must be complemented by a much more bottom-up, instantiated regulatory commitment.

These examples of stewardship operate at a range of different geographical and institutional levels ranging from the supranational (WHO) to the regional (Scotland). Furthermore, the definitions embody different normative aspects and therefore emphasize different values, some of which may conflict. First, the Presidential Commission speaks of responsibility in stewardship. Similarly, the EOSC discusses process rather than actors, which leaves open the question of who will take on the various responsibilities. However, this emphasis on responsibility in process might also point to the need for prudence when dealing with data. Second, in contrast with these examples, the WHO approach to stewardship recognizes a regulatory actor who already has a mandate of stewardship, and is understood as dealing with competing interests. This seems to be a call for ownership of that existing role, to perform it competently. This version of stewardship may also include broader strategic elements, such as assessing public interest claims made on behalf of particular pieces of research and entire research programs. Third, Jansen seems to point to a much deeper kind of stewardship that involves stewards balancing the two important, and sometimes conflicting, values of beneficence and justice. Finally, Thomasma, mentioned previously, describes a steward as a facilitator, for whom the aim and value of the role are far more clearly defined as “one person helping another” to achieve a desired aim. Developing accurate accounts of these normative roles will involve careful teasing out of the varying capacities and responsibilities of the different actors involved in health research regulation.

These diverse roles necessarily embody certain values and responsibilities. These include, but are not restricted to, health promotion, trust, care, guidance, and support. At the same time, it must be acknowledged that these (1) vary considerably depending on context, (2) are potentially and problematically conflicting, and (3) are question begging in terms of the values and responsibilities in play, upon whom the role of stewardship might fall, and with which corresponding tasks.

Overarchingly, however, these examples from the literature do suggest that stewardship is about guiding others with prudence and care across one or more endeavors—without which there is risk of impairment or harm—and with a view to collective betterment. In the sections that follow, we examine what stewardship can mean in regulatory practice across a range of areas in health research, and whether it can constitute an ethical duty for actors engaged in regulatory practice. However, as we also argue, to date, regulatory stewardship is an “invisible” yet crucial component of regulatory frameworks.

## The Invisibility of Regulatory Stewardship

In an increasingly complex regulatory landscape—reflected in the growth of statutes, case law, and guidelines directed at various stakeholders in research endeavors— much has been written about the challenges in navigating the regulatory thicket.^17^ There have been frequent calls for the need to reduce regulatory burden^18,19^ and to achieve proportionality and equivalency in research review and oversight.^20,21^ Those leading these calls include the Academy of Medical Sciences in the United Kingdom, which has released several widely cited reports calling for regulatory streamlining, notably by the adoption of principles such as the following:

1) to safeguard the well-being of research participants;
2) to facilitate high-quality health research to the public benefit;
3) to be proportionate, efficient and coordinated; and
4) to maintain and build confidence in the conduct and value of health research through independence, transparency, accountability, and consistency.^22^

The case for regulatory streamlining was based on a premise and experiences of “a healthcare culture that fails to fully support the value and benefits of health research,” and this led to the creation of the unitary Health Research Authority in the United Kingdom in 2011, whose tripartite mandate is to promote and protect the interests of patients, streamline regulation, and promote transparency in health and social care research. Similarly, efforts to put streamlining into practice are reflected in the United States, where in 2012, Congress passed the Food and Drug Administration (FDA) Safety and Innovation Act that gave authority to the FDA to develop agreements with foreign regulatory authorities for mutual recognition of drug inspections. This culminated in March 2017 in an exchange of letters between the United States and the European Union allowing regulators to rely on their respective good manufacturing practice inspections of pharmaceutical manufacturing facilities, and thereby “enable the FDA and EU to avoid the duplication of drug inspections, lower inspection costs and enable regulators to devote more resources to other parts of the world where there may be greater risk” (press release, March 2, 2017).^23^

Despite these efforts to clear the regulatory thicket and improve pathways for research to proceed efficiently, far less attention has been paid to the contributions, roles, and responsibilities of the full gamut of actors, agents, and institutions who bring about efficient and effective health research within regulatory frameworks, whether such frameworks are viewed as streamlined or ostensibly cluttered. At first glance, it might seem irrelevant to consider that the principal direct beneficiaries of streamlined regulation in this area—researchers—would need assistance in working through the thresholds of regulatory approvals (especially when they are simplified). However, a recent *Nature* article has pointed out that when it comes to regulatory burden on areas such as working with personal health data, researchers “do not have to face the challenge alone.”^24^ Even in a regulatory environment characterized by simplified or streamlined processes and innovative services that improve approval times (or other benchmarks), challenges remain in trying to work through these processes and arrive at the destination of regulatory approval, research commencement, and research output. These challenges are faced not just by researchers, but also regulators themselves, not to mention sponsors, funders, research participants, and the public at large.

This begs a series of connected questions: aside from researchers, on whom does regulatory burden fall, who should work towards its mitigation, and how?

In this article, we do not aspire to answer the empirical dimensions of the questions that were raised. Rather, we suggest that there is a crucially important—but currently largely invisible—role being played in regulatory stewardship in the delivery of objectives such as proportionality, improvement in efficiency, and streamlining of health research regulation. If these are desirable ends (and such a normative question is worthy of further debate in another article), then we posit that this role must be made explicit and given wider recognition and effect, both in law and in practice. This is true not only in the context of data-intensive science, as the *Nature* example suggests, but also across the entire health research endeavor.

Achieving further clarity on what stewardship involves and implies is imperative; otherwise, important potential conflicts of interest (among researchers, institutions, regulators, and sponsors) will remain masked. Both the diversity of the geostructural levels and the diversity in the values embedded throughout point to the need to further unpack what is meant by regulatory stewardship in preexisting conceptualizations and, eventually, what it might look like as the role is more clearly made visible.

Our analysis^25,26^ suggests that these questions require looking beyond formal regulatory infrastructures, and also beyond state-regulated practices, to reveal the complete picture. In making this claim, we argue that there is a central role being played within and throughout health research regulation in the guise of regulatory stewardship. At present, regulatory stewardship appears to embody a dual role of protecting important human interests, such as those of research participants, while at the same time promoting core public goods associated with health research, such as the optimal facilitation of research ethics review and access to research data and other materials.^27^ Although, from a normative perspective, it is possible to support both of these objectives and to envision them working together, it is also important to recognize that they might produce a normative conflict in practice. We address the possible resolution of any tensions in due course; first, we consider instances of where models of stewardship might be found, and extrapolate from these examples what might be solid foundations, values, and principles that support the role(s) articulated.

### Examples of Regulatory Stewardship in Action

Given the focus of this article on regulatory stewardship, this section provides a range of examples from various regulatory environments that demonstrate this role in action.

An overview of research ethics services and associated literature reveals examples of actors already performing regulatory stewardship in either one or both of the ways already outlined. One such example are scientific officers in Scotland’s Research Ethics Service.^28^ These are uniquely qualified individuals attached to the National Health Service (NHS) research ethics committees in Scotland, providing researchers with guidance and support on a variety of matters, including compliance with correct documentation and conformity with legal requirements, all of which could impact the success of their ethics application and their research as a whole. At the same time, scientific officers help guide research ethics committee members in evaluating research applications, particularly when it comes to understanding the regulatory context of a given application. Another British example comes from the Health Research Authority (HRA), which has employed officers known as application managers to help researchers navigate through “complex cases” that straddle regulatory regimes; for example, involving multiple domains such as data, tissue, and devices, and will continue to do so until its streamlining HRA approval mechanism is rolled out for all studies.^29^

Another similar example is NHS research ethics committees themselves. As one of us (E.S.D.) has uncovered in empirical work, ethics committee members, individually and as a group, see themselves as providing a kind of pastoral support to researchers. Yes, they serve to protect the rights, interests, and welfare of research participants, but equally, they feel as though they serve to promote ethical research by working with researchers to secure ethics approval. To be clear, the stewardship practiced by ethics committee members is not necessarily direct and deliberate (and they cannot write an application for a researcher), but through nudges, comments, and responses to queries, members help assuage or even persuade research applicants to improve the quality of their research design or to work around a false roadblock in law (e.g., a misinterpretation that data protection law or adults with incapacity law is stricter than it really is regarding research).

This is seen especially by actors in greater positions of authority within a committee, such as ethics committee chairs or managers (i.e., administrators) who have closer contact with researchers. As one interview participant (a health research regulator) stated: “Medical research is hard. We see 6,000 applications a year for medical research; it is hard, and we need to be helping these people realise their ideas rather than just being what’s seen as a bureaucratic block at the beginning of something that is a very long process.…It’s providing the support to enable people to realise their goals on an ongoing basis,…working in partnership with other people.”^30^ Similarly, the chair of a research ethics committee explained that: “Between meetings, I get lots of correspondence from researchers seeking help and asking for advice. And I’m absolutely happy to do that, because it helps to create the right environment.… Ultimately we all want the same thing, don’t we? We want high quality good research that’s going to make a difference to people’s lives.…We all want that. So, yeah, I try and work with researchers as much as I can.”^31^

A prominent example of a similar attempt to guide researchers is UK Biobank (UKB), which has been funded with more than £350,000,000, inter alia by Wellcome and the UK Medical Research Council. This is with the objective of establishing a resource of human tissue and data involving more than 500,000 participants. A key feature of the governance of UKB is the existence of its Ethics and Governance Council (EGC), designed to act as a “critical friend” to UKB, and it is the responsibility of UKB itself to act as a steward of the resource to promote access for “health-related research.” A stated objective in its Ethics and Governance Framework is to preserve and enhance the resource for the common good of both the research community and future generations.^32^ There is not, however, any explicit acknowledgement in access procedures of the role of front line members of the access team in guiding applicants through the process.^33^ This is a further example of the invisibility of the stewardship role. Note, too, that the frontline officials within UKB as institutional actors have a different role to the oversight and advisory function of the, EGC which stewards the entire endeavor within the broad parameters of obligations to participants and society alike. Any tensions between the roles will be revealed through public reporting of the work of the EGC via the publication of its minutes.

Further afield, the 2015 report of the Council of the Canadian Academies on accessing health and health-related data states:

the Panel found a marked shift among the six best practice entities from a “data custodianship” model, in which holding and securing data are emphasized to the exclusion of other considerations, to a “data stewardship” model, in which enabling access is a core institutional objective proportionately balanced with protecting privacy. The balance is achieved through good governance, which encompasses the definition of an entity’s purpose, objectives, values, and policies.^34^

Although other parameters of good governance are also important—such as transparency, accountability, regulatory effectiveness, and quality—the emphasis here is on the role of stewardship itself in delivering these objectives.

Stewardship has even been put on a legal basis. In New Zealand, section 32 of the State Sector Act 1988 defines “stewardship” as the “active planning and management of medium- and long-term interests, along with associated advice.” The Act declares that: “The government expects regulatory agencies to adopt a whole-of-system view, and take a proactive, collaborative approach to the monitoring and care of the regulatory systems within which they have policy or operational responsibilities.”^35^ Although not explicitly stated, recent reforms in the United Kingdom health research sector appear to do something similar. For example, under the Care Act 2014, it has now been confirmed as a matter of law that health research regulatory agencies have responsibilities not just to protect research participants’ interests, but also to promote ethical and safe research.^36^

It is important to point out that this range of appeals to “stewardship” demonstrates it to be a heterogeneous concept. In the examples of the UK Biobank EGC and the legislative basis from New Zealand, the role appears to be more one of guiding toward particular ends, rather than merely facilitation of access to data or ethics review, as the gatekeeper role of research ethics committees and UK Biobank management would suggest.^37^ This in turn can be contrasted with the examples of the HRA application managers and scientific officers who tend to walk more with researchers on the approvals journey. Nonetheless, what is common to all of the examples is the articulation of a valued end-point in the form of a recognized public good and its association with a responsibility to support other actors toward the realization of such a public good. In the health research context, the public goods in play are numerous and well recognised; they include individual and public health benefits, the promotion of wider social value from research (such as the deepening of human understanding), the redressing of social injustices, and even health/wealth generation. Thus, in the research regulation context one might contrast:

• **State stewards** (acting in a manner deemed to contribute to the public interest; e.g., as established by law),
• **Institutional stewards** (acting like gatekeepers to further institutional objectives),
• **Operational stewards** (acting like ushers through the complexity of established procedures; e.g., access processes),
• **Ethics stewards** (acting with a stated mandate of protection first then the promotion of ethically sound, scientifically robust research after due deliberation).

This is not to suggest that these roles are necessarily always mutually exclusive in remit or value. Still, by seeking to clarify these, we assist in highlighting those values that are exclusive to a role and not to be mixed; we differentiate those that require more or less ethical responsibility, and we highlight those where there might be gray, and potentially dangerous, areas of conflict. Thus, although there is doubtless much activity occurring under the broad rubric of stewardship, these specific features are currently lost in the noise surrounding regulation and governance, and the associated benefits are being obscured as a result.

Even if this is recognized, however, it does not provide sufficient normative grounding to link the identification of well-accepted public goods with any claim that stewardship ought to be promoted as a means to bring about those public goods. Still, we can nonetheless seek to unpack the ways in which these uses of stewardship have been connected to public goods and other socially valued ends as a first step in building a normative case for stewardship.

## What Might Regulatory Stewardship Look Like?

Our examples of stewardship in action are necessarily speculative, partly because our claim is that the role is largely invisible. This makes it more difficult to assess whether there is any added value arising from regulatory stewardship. Moreover, to the extent that there is demonstrable value, the current climate means that the nature and scope of its contribution is greatly underappreciated. As a result, the precise role of effective regulatory stewardship requires a novel conceptualization of what is at stake and what is required.

Unpacking the language of “stewardship” is important. As the previous New Zealand example suggests, it is about “a proactive, lifecycle approach to the monitoring and care of the regulatory regimes.” We see something similar in the United Kingdom under the Care Act 2014, mentioned previously, which requires many regulatory agencies to work together to protect research participants while simultaneously promoting research. But these agencies cannot do this alone. In making our claim, we suggest that all parties seeking to engage with, and in, health research and its regulation must adopt a similar proactive attitude toward their behaviors. Therefore, for researchers, this might mean ensuring that they are sufficiently *au fait* with ethical and legal issues related to the research that they wish to conduct in order to engage meaningfully and in a timely fashion with ethics committees, regulators, and publics. As a minimum, it requires that applications explicitly and in an accessible manner, articulate how the public interest will be promoted through research.

It would follow also from this that researchers must be trained in, and made aware of, this central role in making (good) research happen. As a minimum, this would require researchers to acknowledge their role in contributing to streamlined regulation by responsible discharge of duties to work with regulators effectively. Regulatory stewardship can also be present in peer-to-peer interactions; for example, where researchers’ experience of a particular regulatory mechanism is disseminated to colleagues.^38^ It is crucial that individuals and institutions do not “outsource regulation”^39^ as a matter to be handled by others, perpetuating a culture of Them and Us and an unhelpful dynamic of seeing regulation as a hurdle to be overcome. This is not to suggest that regulatory regimes should not be reformed, but a failure to reveal and explain some of these crucial roles within regulatory schema, runs the risk not only of ignoring achievements to date, but also of directing calls for regulatory and legal reform in entirely the wrong direction.

## Key Features of Regulatory Stewardship

For us, key features from the previous examples include the following.

• The decoupling of stewardship from the state as principal actor in health research regulation, particularly when the state is the source of legal rules and proscribed action in the form of “hard law” regulation; the point is that there are stewardship actors who can and do play a stewardship role in delivering effective health research governance *in addition to* any regulatory functions that might be in operation;
• Relatedly, there is the importance of appreciating that individuals or assemblages of people, not just regulators or their agents, can deliver regulatory stewardship. Empirical evidence has suggested that effective regulation is often only “instantiated” through practice;^40^ that is, it emerges as a product of genuine cooperation of regulators and a range of other actors, including researchers themselves; there are, therefore, good reasons to ask whether efficacy might be improved further still by new actors charged with responsibilities to achieve precisely such ends;
• Regulatory stewardship does not seem to require a legal basis or authority in order to play a role; this helps to explain why it might occur within very informal or even invisible frameworks;
• The ongoing challenge of understanding whether there is a complementary role that is being played by stewards in addition to more formal regulation; for example, in negotiating uncertainty about the relevance of particular legal regimes or rules within regulatory frameworks and finding ways through; and
• The importance of contrasting a concept such as “stewardship” with that of “custodianship”; whereas the latter embodies a largely protective notion in everyday language, the term “stewardship” can be understood as more naturally reflecting the dual roles we posited previously; namely, not simply acting in a protecting capacity, but also promoting the pursuit of clearly identified ends, which in the health research context include the public interest of delivering ethically robust, scientifically sound research. Notwithstanding, this tells us nothing about how to resolve any tensions that might arise in practice when attempting to achieve both roles in a setting that sets them against each other. The silent shift toward a dual role approach, as demonstrated by the Care Act 2014 and the Council of the Canadian Academies report examples (cited previously), must not pass unnoticed or elide policies and practices that foresee and anticipate possible tensions and ways to resolve them.

Accepting this last caveat, we suggest that given all of the important roles that regulatory stewardship is clearly being asked to play, there are good reasons to believe that it adds value to existing (legal) regimes of health research regulation, and as a minimum, that this is a proposition that merits further (empirical) enquiry. Normatively, and crucially, we propose that regulatory stewardship is a responsibility for all actors engaged in health research regulation. The failure to see regulation as an inherently collective responsibility will stand in the way of the optimization of effective regulation. It requires both individuals and institutions, and researchers and regulators alike, to commit to the common goal of progress in human health and well-being.

Taking the example of data and tissue stewards, this responsibility might mean that roles are committed to making resources actively available for research, including not just quality assurance, but also transparent explanations of the contexts in which data and tissue will be available for research. For regulators and ethics and governance managers, it might mean appointing dedicated staff to assist researchers and others to maneuver complex regulatory regimes. For example, the Scottish Health Informatics Programme identified the central role of research coordinators to undertake a brokering function between the researcher and regulator communities.^41^

## Recognizing Potential Limitations Beyond Reality and the Common Interest

We acknowledge certain potential limitations with the regulatory stewardship model. For example, it has been stated that stewards “see properties [in research] not limited to one research field.”^42^ However, this role cannot, and should not, be reduced to a single actor category; that is, to some new form of regulator (or a narrowly defined regulatory role), potentially a new regulatory burden. The risk of creating an actor category is that the role is seen as someone else’s responsibility and effectively someone else’s problem. We argue that stewardship represents and encourages a collective state of mind and action. Even so, this claim to diffuse action throughout the regulatory environment carries its own challenges: who has the authority to act, who is accountable, how does the concept of stewardships interact with other values in health research regulation, such as transparency and fairness, and, ultimately, who is liable? These are all reasonable questions that our analysis throws into sharp relief in making the invisible, visible. The answer, in part, is to avoid seeing this responsibility as part of formal regulatory structures. If the “regulatory space” must be filled, then this is a call to fill it with institutions that support and clearly direct actors operating within that space with clear expectations about their attitudes and behaviors.

At the same time, the crucial watchword of *proportionality* in health research regulation must not be overlooked. Therefore, two principal challenges in giving effect to regulatory stewardship are to address the questions of *who* takes on this role, and *how* the role is to be executed effectively? We have argued elsewhere that the realization of social value from health research is a processual endeavor; that is, that it occurs over time and across numerous regulatory thresholds, from initial ethics review and approval to subsequent publication of research findings and their uptake into practice.^43^ This in itself presents considerable challenges for any stewardship role, because the regulatory landscapes and actors are multiple, overlapping, often disconnected, and sometimes entirely bespoke (as in the context of complex research protocols that involve a range of regulatory objects such as data, tissue, or embryos). The need for proportionality within each of these regulatory silos is now well recognized, but what is less clear and far less appreciated is the unmet need for proportionality across regulatory silos and sectors. It is precisely this unmet need that strengthens any case to embolden a role for regulatory stewardship, tempered by the need always to envision and enact this in ways that only add value and not further regulatory burden.

## Conclusion

We contend in this article that regulatory stewardship is present within health research regulation, but that it is currently largely invisible and, therefore, its potential is underappreciated. We attempt herein to make the implicit explicit, and to reveal some of the normative bases for seeing this as a valuable feature of regulatory landscapes. This is an important start, but it is also only a partial picture. The reality is that regulatory stewardship already happens. We posit that stewardship has a role across all sectors of research, and includes the bridge between the clinic/research divide. Equally, we recognize the risk that by recognizing and formalizing this, it could become another example of outsourcing responsibility. This risk can only be avoided if the charge is taken up that researchers themselves also see regulatory stewardship as part of their role, understand its value, and are offered the necessary support in order to fulfil the associated responsibilities that arise out of the stewardship role. For these reasons, we commend the recommendations of the British Academy/Royal Society and the HLEG, as well as the call of the European Council, and we propose this vital complementary responsibility of *regulatory stewardship*. This is not about regulation as authority, but rather about regulation as a part of a community of common and collective interest.

## Notes

1. High Level Expert Group on the European Open Science Cloud. A Cloud on the 2020 Horizon: Realising the European Open Science Cloud: first report and recommendations, 2016; available at https://www.eudat.eu/sites/default/files/HLEG%20EOSC%20first%20Report.pdf (last accessed 28 Apr 2017).

2. Council of the European Union. Council conclusions on the transition towards an Open Science system. 9526/16 RECH 208 TELECOM 100, 2016; available at http://data.consilium.europa.eu/doc/document/ST-9526-2016-INIT/en/pdf (last accessed 28 Apr 2017).

3. Editorial. European Open Science Cloud. *Nature Genetics* 2016;48:821.

4. British Academy and the Royal Society. Data management and use: Governance in the 21st century, 2017; available at https://royalsociety.org/∼/media/policy/projects/data-governance/data-management-governance.pdf (last accessed 7 July 2017).

5. See note 1, High Level Expert Group on the European Open Science Cloud 2016.

6. Nuffield Council on Bioethics. Public health: Ethical issues, 2007; available at http://nuffieldbioethics.org/project/public-health/ (last accessed 28 Apr 2017).

7. See note 6, Nuffield Council on Bioethics 2007, at para. 2.44.

8. World Health Organisation. Health systems: Stewardship, c.2017; available at http://www.who.int/healthsystems/stewardship/en/ (last accessed 28 Apr 2017).

9. JansenLA. Between beneficence and justice: The ethics of stewardship in medicine. Journal of Medicine and Philosophy 2013;38(1):50–63.2329133210.1093/jmp/jhs058

10. See note 9, Jansen 2013, at 51.

11. See note 9, Jansen 2013, at 62. Emphasis added.

12. See note 9, Jansen 2013, at 55.

13. See note 9, Jansen 2013, at 55.

14. ThomasmaDC. Stewardship of the aged: Meeting the ethical challenge of ageism. Cambridge Quarterly of Healthcare Ethics 1999;8:148–59, at 157.1006718610.1017/s0963180199802047

15. Presidential Commission for the Study of Bioethical Issues. New directions: The ethics of synthetic biology and emerging technologies, 2010, at 4; available at http://bioethics.gov/sites/default/files/PCSBI-Synthetic-Biology-Report-12.16.10_0.pdf (last accessed 28 Apr 2017).

16. See note 15, Presidential Commission for the Study of Bioethical Issues 2010, at 8.

17. LaurieGT, HarmonSH. Through the thicket and across the divide: Successfully navigating the regulatory landscape in life sciences research In: CloatreE, PickersgillM, eds. Knowledge, Technology and Law. Oxon: Routledge; 2014:121–36.

18. Academy of Medical Sciences. A new pathway for the regulation and governance of health research, 2011; available at http://www.acmedsci.ac.uk/policy/policy-projects/a-new-pathway-for-the-regulation-and-governance-of-health-research/ (last accessed 28 Apr 2017).

19. Chief Scientist Office. Delivering innovation through research—Scottish Government health and social care research strategy, 2015; available at http://www.gov.scot/Resource/0048/00488082.pdf (last accessed 28 Apr 2017).

20. TownendD, DoveES, NicolD, BovenbergJ, KnoppersBM. Streamlining ethical review of data intensive research. British Medical Journal 2016;354:i4181.10.1136/bmj.i418127486077

21. DoveES, TownendD, MeslinE, BobrowM, LittlerK, NicolD, et al Ethics review for international data-intensive research. Science 2016;351(6280):1399–400.2701371810.1126/science.aad5269PMC4838154

22. See note 18, Academy of Medical Sciences 2011. Also see the follow-up in 2012 that focused particularly on “streamlining”: Academy of Medical Sciences. Transforming the regulation and governance of health research in the UK, 2012; available at https://acmedsci.ac.uk/file-download/34885-51dd839626e27.pdf (last accessed 28 Apr 2017). The 5 year follow-up report was published in 2017: Academy of Medical Sciences. Regulation and governance of health research: Five years on, 2017; available at https://acmedsci.ac.uk/file-download/14145196 (last accessed 7 July 2017).

23. US Food and Drug Administration. Mutual recognition promises new framework for pharmaceutical inspections for United States and European Union, 2017; available at https://www.fda.gov/NewsEvents/Newsroom/PressAnnouncements/ucm544357.htm (last accessed 28 Apr 2017).

24. LevineA. Biomedical research: Privacy rules. Nature 2016;532:273–4.2708168310.1038/nj7598-273a

25. Taylor-AlexanderS, DoveES, FletcherI, Ganguli-MitraA, McMillanC, LaurieGT. Beyond regulatory compression: confronting the liminal spaces of health research regulation. Law, Innovation and Technology 2016;8(2):149–76.10.1080/17579961.2016.1250378PMC520692828058061

26. LaurieGT. Liminality and the limits of law in health research regulation: what are we missing in the spaces in-between? Medical Law Review 2017;25(1):47–72.2794052510.1093/medlaw/fww029PMC5387842

27. BloomrosenM, DetmerD. Advancing the framework: Use of health data—a report of a working conference of the American Medical Informatics Association. JAMA 2008;15:715–22.10.1197/jamia.M2905PMC258553118755988

28. NHS Research Scotland. Research ethics structure in Scotland, c. 2012–17; available at http://www.nhsresearchscotland.org.uk/services/research-ethics (last accessed 28 Apr 2017).

29. Health Research Authority, c. 2013; available at http://www.hra.nhs.uk/ (last accessed 22 Oct 2016) and https://acmedsci.ac.uk/file-download/34273-5476048647169.pdf (last accessed 7 July 2017).

30. This quote has been extrapolated from 28 in-depth interviews conducted by one of us (E.S.D.) with health research regulators and research ethics committee members in England and Scotland. Interviews were conducted between January and July 2016.

31. See note 30.

32. UK Biobank Ethics and Governance Council. Ethics and Governance Framework, n.d. available at https://egcukbiobank.org.uk/Ethics-and-governance-framework (last accessed 6 Jun 2017).

33. UK Biobank. Access procedures: Application and review procedures for access to the UK Biobank Resource, 2011; available at http://www.ukbiobank.ac.uk/wp-content/uploads/2012/09/Access-Procedures-2011.pdf (last accessed 6 Jun 2017).

34. Council of the Canadian Academies. Accessing health and health-related data in Canada, c. 2011–16; available at http://www.scienceadvice.ca/en/assessments/completed/health-data.aspx (last accessed 22 Oct 2016).

35. State Sector Act 1988; available at http://www.legislation.govt.nz/act/public/1988/0020/latest/DLM129110.html (last accessed 28 Apr 2017).

36. Care Act 2014; available at http://www.legislation.gov.uk/ukpga/2014/23/contents/enacted/data.htm (last accessed 28 Apr 2017).

37. GuilleminM, GillamL, RosenthalD, BolithoS. Human research ethics committees: examining their roles and practices. Journal of Empirical Research on Human Research Ethics 2012;7(3):38–49.2285014210.1525/jer.2012.7.3.38

38. National Data Guardian for Health and Care. Review of data security, consent and opt-outs, 2016. Recommendations 2 and 8; available at https://www.gov.uk/government/uploads/system/uploads/attachment_data/file/535024/data-security-review.PDF (last accessed 28 Apr 2017).

39. Kenny C. Making a mess professionally. Good Doctors, 2016; available at https://www.gooddoctors.org.uk/making-a-mess-professionally/?platform=hootsuite (accessed 22 Oct 2016).

40. StephensN, AtkinsonP, GlasnerP. Documenting the doable and doing the documented: bridging strategies at the UK Stem Cell Bank. Social Studies of Science 2011;41:791–813.2240042010.1177/0306312711423306

41. SethiN, LaurieG. Delivering proportionate governance in the era of eHealth. Making linkage and privacy work together. Medical Law International 2013;13:168–204.2463456910.1177/0968533213508974PMC3952593

42. See note 3, Editorial 2016.

43. Ganguli-MitraA, DoveES, LaurieG, Taylor-AlexanderS. Reconfiguring social value in health research through the lens of liminality. Bioethics 2017;31;87–96.2806042910.1111/bioe.12324PMC5244658

